# Number word structure in first and second language influences arithmetic skills

**DOI:** 10.3389/fpsyg.2015.00266

**Published:** 2015-03-17

**Authors:** Anat Prior, Michal Katz, Islam Mahajna, Orly Rubinsten

**Keywords:** L1, L2, bilingualism, number processing, addition

## Abstract

Languages differ in how they represent numerical information, and specifically whether the verbal notation of numbers follows the same order as the symbolic notation (in non-inverted languages, e.g., Hebrew, “25, twenty-five”) or whether the two notations diverge (in inverted languages, e.g., Arabic, “25, five-and-twenty”). We examined how the structure of number–words affects how arithmetic operations are processed by bilingual speakers of an inverted and a non-inverted language. We examined Arabic–Hebrew bilinguals’ performance in the first language, L1 (inverted) and in the second language, L2 (non-inverted). Their performance was compared to that of Hebrew L1 speakers, who do not speak an inverted language. Participants judged the accuracy of addition problems presented aurally in L1, aurally in L2 or in visual symbolic notation. Problems were presented such that they matched or did not match the structure of number words in the language. Arabic–Hebrew bilinguals demonstrated both flexibility in processing and adaptation to the language of aural–verbal presentation – they were more accurate for the inverted order of presentation in Arabic, but more accurate for non-inverted order of presentation in Hebrew, thus exhibiting the same pattern found for native Hebrew speakers. In addition, whereas native Hebrew speakers preferred the non-inverted order in visual symbolic presentation as well, the Arabic–Hebrew bilinguals showed enhanced flexibility, without a significant preference for one order over the other, in either speed or accuracy. These findings suggest that arithmetic processing is sensitive to the linguistic representations of number words. Moreover, bilinguals exposed to inverted and non-inverted languages showed influence of both systems, and enhanced flexibility in processing. Thus, the L1 does not seem to have exclusive power in shaping numerical mental representations, but rather the system remains open to influences from a later learned L2.

## Introduction

Bilingual speakers have control of two languages and hence raise important questions regarding language and cognitive representations and processing. Such questions include the degree to which two languages are represented or processed independently versus interactively (e.g., Kroll and Stewart, 1994; Costa, 2005), as well as the impact of language on cognitive representations more generally. The fact that languages differ in their structural properties provides an elegant method of investigating to what degree cognitive representations, in the current case in the numerical domain, are uniquely shaped by the native language (L1) or rather remain open to influences from a later acquired second language (L2). This question has been previously addressed in the domain of semantic/conceptual representations. Thus, Jiang (2002, 2004) proposed a model according to which the conceptual system is mostly shaped by the L1, except in highly proficient bilinguals. In contrast, Degani et al. (2011) demonstrated sensitivity of semantic processing to L2 lexical properties, even in unbalanced moderately proficient bilinguals (see also Cook, 2003; Laufer, 2003; Wolff and Ventura, 2009). The role of the L1 vs. the L2 in shaping representations and processing has also been investigated in the numerical domain (Gelman and Butterworth, 2005), again leading to conflicting results.

For example, Macizo et al. (2010) showed that the processing of number words in one language was not modulated by the way bilinguals processed number words in their alternative language, which differed in the structure of number words. In contrast, it has been consistently shown that even proficient L2 speakers resort to their L1 to perform mathematical operations (e.g., Spelke and Tsivkin, 2001). In addition, Salillas and Carreiras (2014) recently demonstrated specific influences of the structure of the language in which early math instruction occurred on the processing of numerical information in highly proficient balanced bilinguals.

These conflicting results raise the question to what degree people can learn to process numerical information according to the structure of their L2, when it differs markedly from that of the L1, specifically when they are highly proficient L2 speakers. The current work extends this controversial line of research by investigation the impact of both the L1 and the L2 on numerical representation and calculation in highly proficient bilinguals, whose languages differ in the structure of number words.

This question is of central importance, because arithmetic processing in monolinguals is closely linked to language (Dehaene, 1992; McCloskey, 1992; Campbell, 1994; De Smedt et al., 2010; Archibald et al., 2013 ). Thus, it has been shown that the four basic operations (addition, subtraction, multiplication, and division) are learned in school with different emphasis on quantity manipulations and on linguistic skills (Dehaene and Cohen, 1995; Delazer et al., 2006; Ischebeck et al., 2006), with multiplication and addition being retrieved from verbal memory but subtraction and division requiring manipulation of quantities (e.g., Zhou et al., 2006). In general, it has been suggested that with advanced age and practice, counting and using quantity knowledge to achieve an outcome is replaced as the strategy of choice by memory retrieval, similar to the way words are retrieved from the verbal lexicon, at least in cases of addition and multiplication (e.g., Delazer et al., 2006; Ischebeck et al., 2006). However, it should be noted that Pesenti et al. (2000) and Venkatraman et al. (2005) did not find any language-related frontal activations for symbolic exact arithmetic involving simple addition problems, suggesting that different strategies, other than retrieval from verbal memory, may be in use.

These findings lead to fascinating questions concerning the cognitive mechanisms underlying mathematical operations in proficient bilinguals, especially when information is presented in the L2. For example, when doing arithmetic in the L2, do bilinguals rely on the linguistic structure of that language, and how do these processes interact with the L1? These questions address the fundamental issue of whether human cognitive capacities related to the L1 and the L2 employ a shared or independent cognitive system. Numerical knowledge acts as a natural and ecological laboratory for the study of L1/L2 interactions, as bilinguals have three sets of symbols to represent the same semantic concept: written or symbolic digits (3), L1 number words (e.g., *shalosh* in Hebrew or *talate* in Arabic) and L2 number words. This makes it possible not only to study translation from L1 to L2 and from L2 to L1 but also from a common semantic meaning to written or verbal forms in either language. In the current study we extend the examination of bilingual cross-language interaction by asking whether the structure of number words in one language is modulated by the way bilinguals process numbers in the alternative language.

Languages differ in the structure of number words and how they are used, and such differences can shape the way in which speakers of a certain language process numbers. Thus, several studies set out to examine the idea that variability in mathematics performance may be related to differences in the cognitive organization of numbers that is affected by number–word characteristics of a language. Thus, number words in Chinese, Japanese, and Korean are congruent with the traditional base 10 numeration system, such that the spoken number corresponds exactly to the implied quantity represented in the written form (i.e., the number 49 is written in character symbols as four-10s-nine). Number words in English, on the other hand, may lack the elements of 10s and ones that are contained in them (i.e., the number 12, twelve). Miura et al. (1988) found that whereas first grade native speakers of English preferred to use a collection of unit blocks to represent numbers, speakers of base-10 languages more frequently used a construction of 10s and ones, in correspondence with the linguistic structure (see also Fuson and Kwon, 1992; Miller et al., 1995; Geary et al., 1996).

In a more recent study, Colomé et al. (2010) compared Italian and Catalan speakers. Italian is a base-10 language while Catalan number-words are constructed by combining multiples of 20 with units or with teens (e.g., the verbal representation of 35 is “twenty and fifteen”). Their results showed a consistent difference between the two groups in their preference toward a certain number–word structure when solving problems verbally and when typing their answers in Arabic numerals. The researchers concluded that language differences in the structure of number–words play a role when solving addition problems.

The current study focuses on the property of inversion, coined by Zuber et al. (2009) to describe the situation when the order of the symbolic and verbal notation of a number are inverted. For example, the number “25” in inverted languages is pronounced as “five and twenty.” The inversion property affects all two-digit numbers from 21 to 98, repeats for the 10,000s, and is a feature of various languages such as Arabic, Danish, Dutch, and German. There is evidence showing that children who speak languages with inversion have difficulty in basic numerical transcoding tasks, namely the ability to translate numerals from one form to another, such as the Arabic notation “*27”* to verbal notation “*seven and twenty”* (e.g., Pixner et al., 2011; Imbo et al., 2014). Difficulty in such tasks, probably due to the multiple inversions required in representing two-digit numbers, consequently leads to working memory overload (Zuber et al., 2009).

In adults, Brysbaert et al. (1998) tested the theory that numerical addition is based on language processes by comparing French and Dutch-speaking participants solving addition problems such as 20 + 4 and 4 + 20 (unit + decade) and 21 + 5 and 5 + 21 (unit + decade-unit) presented either as Arabic numerals or as number words. The French participants solved operations like “20 + 4” and “21 + 5” faster than their counterparts in the other order, both when presented with Arabic digits and with number words. The Dutch participants differed in their performance; operations like “20 + 4” were preformed faster in this order only when presented in the numeric format, but no differences between “20 + 4” and “4 + 20” were found for the verbal format. When the operation consisted of decade-unit + unit (21 + 5), they were faster to answer in the inverse order (5 + 21). The results demonstrated some differences in preference for order of operations based on language when the questions were presented in written verbal form and the participants were asked to respond verbally. On the other hand, the two groups did not differ significantly in their responses when asked to type the answers numerically. The authors concluded that the numerical system is largely autonomous of the language system.

However, a later study by Nuerk et al. (2005) compared English and German speakers’ performance in magnitude comparison. Two numbers were presented above each other on a computer screen and participants were asked to determine which number was larger. This study showed influence of the inversion property in a unit-decade compatibility effect. This compatibility effect is found when two-digit Arabic numbers are compared, such that cases where separate decade and unit comparisons lead to the same decision (e.g., 32_47; in this case 3 < 4 and 2 < 7) are processed faster than incompatible trials (e.g., 37_52; in this case 3 < 5, but 7 > 2). According to McCloskey (1992), there may be separate mental number line representations for decades and units which, in turn, may be separately processed in two-digit number comparison. If this is true, comparing a pair of incompatible numbers could be a more difficult and lengthy process than comparing a pair of compatible numbers.

In the above mentioned study, Nuerk et al. (2005) investigated the generality of the compatibility effect by comparing English and German speakers. They found that while for native German speakers the compatibility effect is much larger for large unit distances than for small unit distances, for native English speakers the compatibility effect is larger for small decade distances than that of the German speakers. Moreover, large unit distances and small decade distances led to disproportionately more errors for English participants but not for German participants. The authors therefore concluded that decade distance seemed to determine responses in English speakers, while overall distance was the most important predictor for German speakers, particularly when dealing with written number words. Thus, the lexical representation in a language influences magnitude comparison even when numbers are presented in a non-linguistic format.

A recent study conducted by Macizo and Herrera (2010) strengthens this conclusion by testing Spanish speakers’ number processing when presented with two-digit number words in reverse form (unit-decade order, e.g., five-and-twenty). In each trial, one number word was presented above the other in the center of the screen and the participants had to select the larger of the two numbers. Based on the effects of the decade distance and the compatibility effect, the results showed that only decade distance was a significant predictor for difference in reaction time (RT). The authors concluded that speakers of non-inverted languages have learned a language-dependent process for analyzing written numbers in which decades have a major role regardless of the position in which they are presented experimentally. These findings reinforce the theory that the spoken language does in fact affect the way in which numbers are processed when presented in both numeric and verbal form.

To date, there is only a handful of studies that have taken a close look at number processing in bilinguals who speak both an inverted language and a non-inverted language. These studies have suggested that bilinguals process two-digit number words selectively in their L1 and L2 and that they do not seem to transcode number words from their L2 into Arabic number format. In other words, most studies have found that the processing of number words in the L1 does not influence the way bilinguals process number words in their L2 (Macizo et al., 2010, 2011). Macizo et al. (2010) examined the way Italian/German bilinguals performed a number comparison task by presenting them with compatible and incompatible number–word pairs in their two languages. Participants were faster when presented with compatible pairs than incompatible pairs in German, while they were slower when presented with compatible pairs than incompatible pairs in Italian. The authors concluded that bilingual speakers are not bound to the number-structure of their L1 and the relative reliance on the decade and unit values differ depending on the language of presentation; when processing number–words in an inverted language, they rely on the unit values, and when processing number–words in a non-inverted language, they rely more on the decade values.

A more recent study by Macizo et al. (2011), also investigated between-language influences by comparing Spanish/English and German/English bilinguals’ performance on a number comparison task. Their results show that both bilingual groups presented a reverse compatibility effect when performing the comparison task in the L2 (a non-inverted language) but differed in the way they processed L1 numbers. A reverse compatibility effect was observed in the L1 Spanish task for the Spanish/English bilinguals (an expected pattern for a non-inverted language), and a regular compatibility effect was observed in the L1 German task for the German/English bilinguals (an expected pattern for an inverted language). The finding that bilinguals processed two-digit number words selectively in their L1 and L2 means that bilinguals are influenced by the language of presentation and process numbers according to the expected pattern for each language.

Taking such recent findings into account, the question that remains unanswered is whether or not cross-language influences exist in other numerical processing tasks, namely in arithmetic calculation. We investigate this question by presenting Arabic/Hebrew bilinguals and native Hebrew speakers with addition problems composed both in visual–symbolic notation and in aural–verbal presentation. Similar to Brysbaert et al. (1998) we manipulated the order in which the elements of the addition problems were presented (20 + 5 vs. 5 + 20) such that they did or did not match the structure of number words in the language. To our knowledge, this was the first study to use number words in an aural–verbal format instead of presenting number words in a written format. Thus, the current study will test the influence of language on number processing by examining the effect of the structure of number words in a language on processing addition problems, as well as the susceptibility of speakers of inverted and non-inverted languages to decade and unit numerical values.

We are particularly interested in whether the organization of numerical processing is determined by one’s L1, which in this case is also the language of math instruction, or whether it is open to influences from the L2 as well. If the former is true, the performance of Arabic speakers in both aural–verbal presentation and in visual–symbolic presentation should reflect the inversion property of their L1. However, if the latter is true there are two possible patterns: they might show different preferences depending on the language in which the problem is presented. The second option is that Arabic–Hebrew bilinguals in the current study might show enhanced flexibility in processing, such that they become less sensitive overall to differences between presentations that match inverted or non-inverted structures.

## Materials and Methods

### Participants

Sixty three students from the University of Haifa participated in the study: 31 Arabic–Hebrew bilinguals (22 women, mean age 22) and 32 Hebrew–English bilinguals (20 women, mean age 26). Participants were recruited through flyers and online ads. Participants gave informed consent and were paid 30 NIS an hour (45–60 NIS in total). The study was approved by the research ethics committee of the University of Haifa. All participants included in the study reported no history of language and∖or numerical disabilities.

### Materials

#### Language Experience and Proficiency Questionnaire (LEAP-Q)

The LEAP-Q (Marian et al., 2007) is a computerized self-report questionnaire that gathers information regarding participants’ language background and abilities in all the languages they speak. The questionnaire includes questions regarding age of acquisition of languages, oral and written self-rated proficiency in all the languages a participant speaks, and the percent of time each language is used. The questionnaire was written in Hebrew and all participants were encouraged to ask questions if a portion of the questionnaire was unclear to them.

#### Arithmetic Two-Minute Test

Participants’ mathematical automaticity skills were assessed using the Arithmetic Two-Minute test (Openhin-Bitton and Breznitz, unpublished). This task consists of 80 simple arithmetic calculation problems, including the four basic math operations (addition, subtraction, multiplication, and division). The problems were presented in four columns, 20 problems for each basic math operation. Participants were instructed to solve as many problems as possible, from all four types, in 2 min. Total time, accuracy and correct responses per minute were scored.

#### Working Memory Test

Memory performance was assessed using a computerized N-Back task (Owen et al., 2005), comprised of digit and spatial memory subsets. In both tasks, a sequence of digits or square locations was displayed on the computer screen and participants indicated when the current stimulus was identical to the stimulus that appeared on the previous trial by pressing on the “space” bar. There were 60–75 steps in each task (totaling 135 steps), 15 of which included target stimuli. Each trial started with a fixation point for 250 ms, a black screen for 500 ms, a stimulus for 500 ms, and a black screen for one second. Digit span was assessed using six digits (1, 2, 3, 4, 5, 6), and spatial memory was assessed using six different square locations on the computer screen. Participants could respond once the stimulus appeared or after 1 s. In addition, 5 s breaks were provided every 24 trials.

#### Experimental Task: Verifying Addition Problems

Participants responded to addition problems presented to them in three formats: visual–symbolic (Arabic numerals), aural–verbal in the L1, and aural–verbal in the L2 (see **Table 1**). In order to balance the design, Hebrew speaking participants also completed an aural–verbal block in English, their L2. However, because the structure of number words does not differ between Hebrew and English, this block was not theoretically relevant, and therefore results were not analyzed.

**Table 1:** Demonstration of experimental materials, by problem type and condition.

		Match	Non-match
Correct problems	*Arabic aural–verbal*	Five plus twenty equals five and twenty	Twenty plus five equals five and twenty
	*Arabic visual–symbolic*	5 + 20 = 25	20 + 5 = 25
	*Hebrew aural–verbal*	Twenty plus five equals twenty-five	Five plus twenty equals twenty-five
	*Hebrew visual–symbolic*	20 +5 = 25	5 + 20 = 25
Incorrect decade	*Arabic aural–verbal*	Five plus twenty equals thirty-five	Twenty plus five equals thirty-five
	*Arabic visual–symbolic*	5 + 20 = 35	20 + 5 = 35
	*Hebrew aural–verbal*	Twenty plus five equals thirty-five	Five plus twenty equals thirty-five
	*Hebrew visual–symbolic*	20 + 5 = 35	5 + 20 = 35
Incorrect unit	*Arabic aural*	Five plus twenty equals twenty-four	Twenty plus five equals twenty-four
	*Arabic numeric*	5 + 20 = 24	20 + 5 = 24
	*Hebrew aural*	Twenty plus five equals twenty four	Five plus twenty equals twenty four
	*Hebrew numeric*	20 + 5 = 24	5 + 20 = 24

Although verbal representations in the table are written in English to illustrate the order of elements in the problems, in the actual experiment all verbal materials were recorded in Hebrew or Arabic. In addition, all visual–symbolic problems were presented in Arabic (not Indian) numerals.

Problems were presented with answers, and participants indicated by button press if the equation was correct or not. RT and accuracy of responses were recorded. All critical problems were comprised of the addition of a round decade number and a single unit number (e.g., 20 + 5 = 25). Addition problems were constructed using three numerical ranges (20–29, 40–49, and 70–79). Elements of the problem could be presented such that they matched or did not match the order of number words in participants’ language. The order manipulation was implemented across both aural–verbal and visual–symbolic presentation. Across participants each problem appeared in both the Match and the Non-match condition.

##### Match

The structure of the verbal representation of the problem *matches the structure of number words in the language*; i.e., “five plus twenty equals five and twenty” or “5 + 20 = 25” for Arabic and “twenty plus five equals twenty five” or “20 + 5 = 25” for Hebrew.

##### Non-match

The structure of the verbal representation of the problem *does not match* the structure of number words in the language; i.e., “twenty plus five equals five and twenty” or “20 + 5 = 25” for Arabic, and “five plus twenty equals twenty five” or “5 + 20 = 25” for Hebrew.

For each addition problem *correct* and *incorrect* responses were constructed. Incorrect answers consisted of an error either in the *units* or in the *decades:*

##### Incorrect unit

The wrong answer was in the same decade of the correct answer, but the unit value was different. If the numeral unity was under 5, it was replaced by a number between 5 and 9 at random; if the unit number was above 5, it was replaced by a number between 0 and 4 at random (see **Table 1**).

##### Incorrect decade

The wrong answer shared the same unit of the correct answer, but the decade value was different. Each group of decades was divided into two sub-groups: units under 5 and units above 5. In each sub-group, the decades were changed with a smaller value (minus 1) or greater value (plus 1) at random (see **Table 1**).

Finally, two types of filler addition problems were added to the list. The first type included problems from the second decade (11–19) of similar structure to the critical items. The second type of filler items were problems which did not match the structure of number words in either of the languages; e.g., “twenty three plus four equals twenty seven.” These problems were included in the experiment in order to provide the participants with a list of diversified problems and so that they do not pick up on a pattern of the first two types of problems. The filler problems could also include carry procedures. However, since this type of problems is not relevant for the theoretical questions presented in this study, they were not further analyzed.

When all stimuli were constructed, three comparable lists each containing 96 items were created. Each list included 24 items in the Match condition (12 correct, 6 incorrect Decade, 6 incorrect Unit); 24 items in the Non-match condition (12 correct, 6 incorrect Decade, 6 incorrect Unit) and 48 filler items (24 correct and 24 incorrect). All three lists were orally recorded in Arabic, Hebrew, and English, by a native speaking female of each language, respectively. Each problem and each answer was saved in separate sound files, played consecutively to participants. This allowed randomization of presentation order across participants, and also allowed us to measure response RT from the onset of the answer, leading to more accurate assessment of performance.

### Procedure

The tasks were divided into two 1-hour sessions. The first session included the LEAP-Q, the Two-Minute Test, and the Working Memory task. The second session included the experimental task of verifying addition problems. All computerized tasks were programmed in E-Prime, and the participants sat approximately 60 cm from the screen.

#### Experimental Task Presentation

##### Aural–verbal blocks

Each block started with written instructions in the language of the following block. Participants were instructed to respond as quickly and as accurately as possible.

Addition problems were presented through headphones, and did not appear on the screen, though participants responded using a computer keyboard. Each trial started with a fixation cross for 400 ms, followed by a blank screen for 150 ms, after which the problem was presented aurally while a green dot appeared in the center of the screen. The green dot remained on the screen until the participants responded. Participants used their index finger to press the right key for a correct answer or the left key for an incorrect answer. After responding, a red circle appeared in the center of the screen and participants pressed a key to initiate the following trial, to ensure that all participants had the same allotted response time. Each language block included 96 trials, and participants were given two short breaks during the block.

The instructions were followed by a practice block including 18 addition problems (nine problems per language). Participants were given feedback on their performance in the practice block. The experimental block, however, did not provide the participants with feedback on their performance.

##### Visual–symbolic block

Addition problems including answers were presented at the center of the screen. Each trial started with a fixation cross for 400 ms, then a blank screen for 150 ms, after which the addition problem was presented centrally in Arabic numerals until participants responded with the right key if the problem was correct and with the left key if it was incorrect. Responses were followed by a red circle appearing in the middle of the screen, and participants pressed a key to initiate the next trial. The experimental block was preceded by a practice block of nine addition problems, for which participants received feedback.

Arabic speaking participants completed one list aurally in Arabic, one list aurally in Hebrew, and one list visually. Hebrew speaking participants completed one list aurally in Hebrew, one list aurally in English, and one list visually. The assignment of list to presentation condition was counterbalanced across participants, as were the order of visual vs. aural presentation, and the order of L1/L2 within the aural presentation. Within each list, item presentation was randomized for each participant. The 96 items in each list were randomly divided into three blocks, each containing 32 items. Participants were given breaks between blocks.

## Results

### Background Variables

The group performance in the background variables is presented in **Table 2**. The performance of the Arabic and Hebrew speakers was compared in working memory (N-back task), language background (LEAP-Q) and arithmetic abilities (Two-Minute arithmetic task). The Arabic speakers were significantly younger than the Hebrew speakers, *t*(60) = 5.32, *p* < 0.001. However, there was no significant difference between the groups in years of education (*p* = 0.15).

**Table 2:** Means (SD) of participant characteristics.

	Native Arabic speakers *N* = 31	Native Hebrew speakers *N* = 32
Age*	21.65 (2.4)	25.73 (2.9)
L1 self-rated proficiency	9.71 (0.49)	9.85 (0.31)
L2 self-rated proficiency	7.71 (1.37)	7.47 (1.88)
L2 age of acquisition	8.90 (1.7)	7.26 (3.15)
Participant years of education	14.63 (1.96)	14.35 (1.7)

*Means significantly different at *p* < 0.01.

Additionally, there was no significant difference between the two groups when performing the arithmetic two-minute test, (*p* = 0.66). In other words, the Arabic speaking participants and the Hebrew speaking participants did not differ significantly in the number of arithmetic problems solved correctly in a two-minute span.

The working memory task, which required the participants to recall numerical and spatial stimulus 1 or 2 steps back, revealed a main effect of participant group, because Arabic speaker had shorter RTs than Hebrew speakers across all conditions, *F*(1,61) = 4.44, *p* < 0.05. However, both groups were equally accurate, again across all conditions, *F*(1,61) = 1.24, *p* = 0.27. Previous research has shown that accuracy in working memory tasks is a more sensitive index of individual differences in working memory (Unsworth and Engle, 2008). Therefore, we do not further analyze the speed differences between the participant groups.

### Experimental Tasks – Addition Problems

In order to address the theoretical issue of the impact of number word structure on numerical processing, we conducted three main comparisons. In the processing of aural–verbal problems we first compared the performance of Arabic speakers in Arabic (the L1, an inverted language) and Hebrew (the L2, a non-inverted language). Then, we compared the performance of Hebrew speaking and Arabic speaking participants in their performance on Hebrew aural–verbal problems. This comparison allowed us to investigate whether speakers of an inverted L1 might process a non-inverted language differently than native speakers of a non-inverted L1. Finally, we compare the performance of the two participant groups on their responses to visual–symbolic problems. An important aspect of the two comparisons across participant groups is that they were based on the exact same stimuli for all participants.

#### Arabic Speakers, L1/L2 Aural Presentation

To compare the performance of native Arabic speakers in L1 and L2, we conducted a three-way repeated-measures ANOVA on accuracy rates, and on mean RTs for correct responses. Within participant variables were Presentation Language (Arabic, Hebrew), Order (Match, Non-match to the structure of number words in the language of presentation), and Correctness (correct, incorrect Unit, incorrect Decade).

In the analysis of RTs, there was a main effect of presentation language *F*(1,28) = 42.5, *p* < 0.001, η = 0.6, because participants were faster to respond to addition problems in Arabic, the L1, than in Hebrew, the L2 (**Table 3**). Although participants were numerically faster to respond to problems that matched the structure of number words in the relevant language (inverted in Arabic, non-inverted in Hebrew), this difference did not reach statistical significance, *F*(1,28) = 2.1, *p* = 0.16. This finding is noteworthy in that it demonstrates that Arabic speaking participants were not sensitive to order of presentation, and regardless of whether they were listening to problems in the L1 or the L2 they were equally able to respond to problems presented in inverted or non-inverted order (see **Table 3**). Finally, the two-way interaction between presentation language and correctness was significant, *F*(2,56) = 19.1, *p* < 0.01, η = 0.7. This interaction is driven by the fact that in Arabic, participants were faster to respond to problems with an incorrect unit, whereas in Hebrew they were faster to respond to problems with an incorrect decade. Because in Arabic the unit information becomes available first in aural presentation (five-and-twenty) whereas in Hebrew the decade information becomes available first (twenty-and-five) this pattern is expected. No other main effects or interactions were significant.

**Table 3:** Mean RTs (SD) for aural–verbal addition problems, by language and by order.

	Native Arabic speakers in Arabic (L1)	Native Arabic speakers in Hebrew (L2)	Native Hebrew speakers in Hebrew (L1)
Inverted	1297 (50)	1697 (60)	1655 (64)
Non-inverted	1313 (66)	1635 (58)	1560 (57)

**FIGURE 1:**
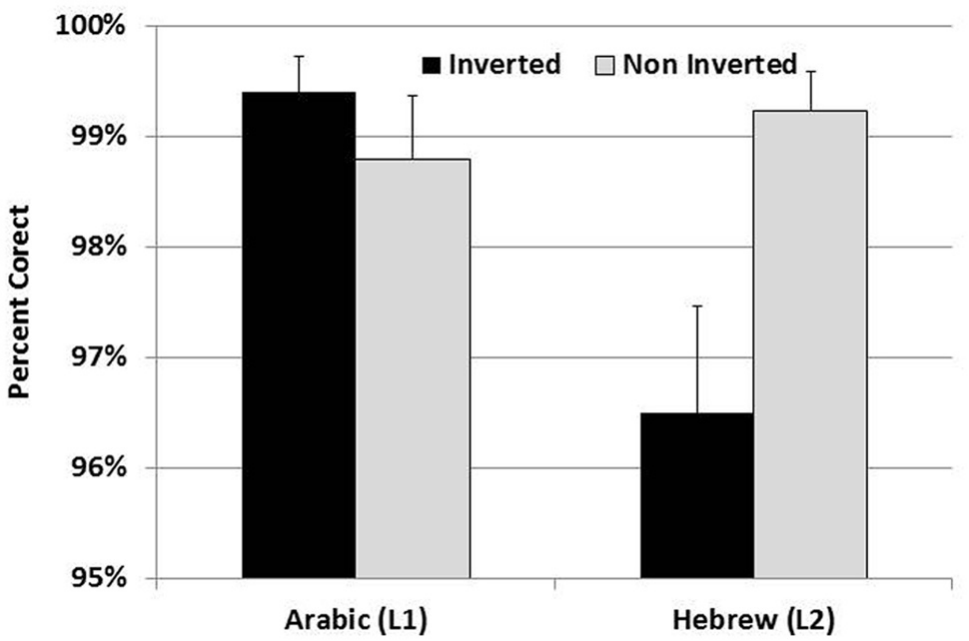
**Accuracy rates of Arabic speakers to aural–verbal addition problems, by language of presentation and order of presentation.** *Note*: Inverted order (5 + 20 = 25) matches the structure of number words in Arabic, but not in Hebrew. Non-inverted order (20 + 5 = 25) matches the structure of number words in Hebrew, but not in Arabic.

In the accuracy analysis there was a significant main effect of presentation language, *F*(1,28) = 4.90, *p* < 0.05, η = 0.15, because participants were more accurate overall in the L1 than in the L2. In addition, there was a main effect of Order, *F*(1,28) = 7.7, *p* < 0.01, η = 0.2, because participants were more accurate to judge addition problems adhering to the structure of number words in the language of presentation, than to problems that did not match the structure of number words (see **Figure 1**). Importantly, the effect of Order was stable across both languages of presentation (namely, the interaction between Order and Language was not significant), indicating that in Arabic participants were more accurate in judging problems presented in the inverted order, whereas in Hebrew they were more accurate in judging problems presented in the non-inverted order. This shows flexibility and adaptability of processing preferences to the language of presentation.

#### Comparing Hebrew and Arabic Speakers on Aural–Verbal Presentation in Hebrew

To compare the performance of native Hebrew and native Arabic speakers in responding to aural–verbal addition problems presented in Hebrew, we conducted a three-way mixed design ANOVA, on reaction times and accuracy (**Table 3**). Within-participant variables were Correctness (correct, incorrect-Unit, incorrect-Decade), Order (Match, Non-match to the structure of number words in the native language), and the between participant variable was native language group (Arabic, Hebrew).

Analysis of RTs to Hebrew aural presentation revealed a significant main effect of Order, *F*(1,58) = 5.9, *p* < 0.05, η = 0.1, because participants were faster to respond to addition problems that match the structure of number words in Hebrew, than to problems that do not match this structure. The two-way interaction between Order and Language Group was not significant, *F* < 1, showing that native Hebrew and native Arabic participants showed very similar patterns of performance and sensitivity to the order manipulation. This finding aligns with the pattern reported above, comparing the accuracy of performance of the native Arabic speakers in Arabic and in Hebrew.

Although native Hebrew speakers, performing the task in their L1, were numerically faster than native Arabic speakers performing the task in their L2 (mean RTs of 1607 and 1637 ms, respectively), this difference was not statistically significant, *F* < 1. The main effect of correctness was significant, *F*(1,58) = 7.1, *p* < 0.05, η = 0.35, because participants were slower to respond to problems with incorrect units (*m* = 1736) than to correct problems (*m* = 1622) or to problems with incorrect decades (*m* = 1551). Again, we interpret this pattern as a result of the time at which information becomes available as the answer to the problem unfolds aurally. No other interactions were significant.

The analysis of accuracy rates again revealed a significant main effect of Order, *F*(1,58) = 6.6, *p* < 0.05, η = 0.1, because all participants were more accurate to judge addition problems that matched the structure of number words in Hebrew than problem that did not match this structure. Crucially, the effect of Order did not interact with Language Group, demonstrating that this preference was shared by both native Arabic and native Hebrew speakers. This is the same pattern that was reported above for the RTs. There were no other significant main effects or interactions.

#### Comparing Hebrew and Arabic Speakers on Visual–Symbolic Presentation

**FIGURE 2:**
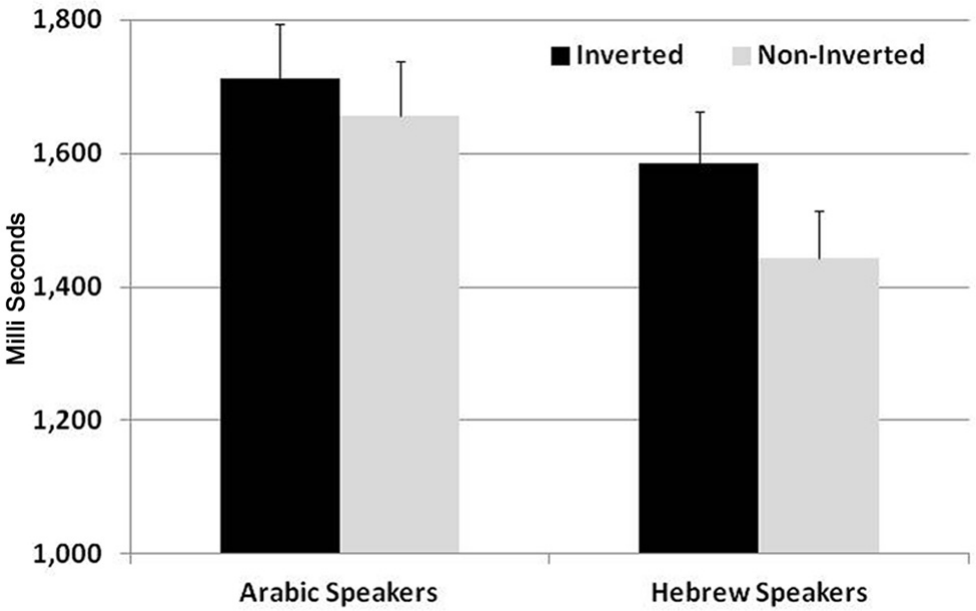
**Reaction times (RTs) to visual–symbolic addition problems.** Inverted order (5 + 20 = 25) matches the structure of number words in Arabic, but not in Hebrew. Non-inverted order (20 + 5 = 25) matches the structure of number words in Hebrew, but not in Arabic.

To compare the performance of native Hebrew and native Arabic speakers in responding to visual–symbolic addition problems, we conducted a three-way mixed design ANOVA, on reaction times and accuracy (see **Figure 2**). Within-participant variables were Correctness (correct, incorrect-Unit, incorrect-Decade), Order (Match, Non-match to the structure of number words in the native language), and the between participant variable was native language group (Arabic, Hebrew).

In the analysis of RTs there was a significant main effect of Correctness, *F*(1,60) = 8.6, *p* < 0.01, η = 0.2. Participants were faster to respond to correct than to incorrect problems. There was also a significant two-way interaction between Order and Language group, *F*(1,60) = 6.7, *p* < 0.05, η = 0.1. Follow up comparisons showed that whereas native Hebrew speakers were significantly faster to respond to problems matching the structure of number words in Hebrew than to non-matching problems [*t*(30) = 2.7, *p* < 0.01], native Arabic speakers did not show sensitivity to the order manipulation, *t*(30) < 1. No other main effects or interactions were significant.

In the analysis of accuracy rates, the only significant finding was a three-way interaction between Order, Correctness, and Language group, *F*(2,120) = 4.2, *p* < 0.05, η = 0.1. Follow up comparisons showed that for Arabic speakers there were no significant effects in accuracy for either Order of presentation or Correctness (all *F* < 1). Conversely, for Hebrew speakers there was a significant interaction between Order and Correctness, *F*(2,60) = 4.3, *p* < 0.05, because they showed lower accuracy rates for problems with incorrect units presented in the non-matching order.

## Discussion

The present study examined whether adult university students show a preference for processing addition problems presented in an order that matches the structure of number words in their native language. Furthermore, we investigated the permeability of numerical processing to the structure of number words in the L2, especially when it differs markedly from the L1. We found that native Hebrew speakers, whose L2 (English) shares the same non-inverted structure of number words as the L1, have a marked preference both in aural–verbal presentation and in visual–symbolic presentation for addition problems presented in an order that matches the familiar structure of number words. Conversely, we found that Arabic–Hebrew bilinguals showed more flexibility in their patterns of performance, though the patterns revealed by the data were somewhat more complex. Thus, when comparing the performance of Arabic–Hebrew bilinguals across their two languages and for visual–symbolic problems, they did not show a preference in RTs for either inverted or non-inverted problems. However, when comparing their performance to that of native Hebrew speakers for aural–verbal problems presented in Hebrew, they did show the same pattern, of a preference for non-inverted over inverted problems. This preference was also apparent in the Arabic–Hebrew bilinguals’ accuracy rates for aural–verbal problems presented in their two languages. Thus, they were more error prone when the structure of the addition problem mismatched the structure of number words in the language of presentation. Therefore, both the possible patterns identified in the introduction are apparent in the performance of the Arabic–Hebrew bilinguals. On the one hand, we found evidence for some adaptation to the language of presentation, mostly in accuracy rates. On the other hand, the Arabic–Hebrew bilinguals also show evidence for enhanced flexibility, expressed as less sensitivity overall to the alignment between the order of presentation of addition problems and the structure of number words in the language.

The current results regarding the effect of order of presentation proved to be quite interesting. Previous findings comparing languages that differ in the structure of number words (Brysbaert et al., 1998; Colomé et al., 2010) support a prediction that speakers of inverted languages should prefer to solve problems that follow the order of inverted number words (unit-decade), while speakers of non-inverted languages would prefer to solve problems that follow the order of non-inverted number words (decade-unit). Colomé et al. (2010), who compared Italian and Catalan speakers, argued that language differences in the structure of number–words play a role when solving addition problems. They reached this conclusion after finding that the differences between the two groups’ preference toward a particular number–word structure remained consistent both when solving problems verbally and when typing their answers on a keyboard.

Brysbaert et al. (1998), who compared Dutch and French speakers, also found that the order of presentation of addition problems, and whether it matched the structure of number words, influenced participants’ performance when asked to respond verbally. Nonetheless, since these results were not replicated when participants typed their answers on a keyboard, the authors concluded that the differences between the two languages were due to a strategic adaptation to verbal output requirements instead of a direct influence of language in the addition stage.

The results of the current study show that whereas the Hebrew speakers followed the expected pattern, showing a preference for problems that follow a non-inverted order, the Arabic speakers were equally facile in responding to visual–symbolic addition problems presented in inverted and non-inverted order. In contrast, in aural–verbal presentation the Arabic–Hebrew bilinguals showed less sensitivity to order of presentation in indices of RT, but were more accurate for inverted problems in Arabic and for non-inverted problems in Hebrew. These findings suggest that the Arabic speakers are flexible and show a shift in language-order preference. In other words, it seems that by being exposed regularly to both an inverted language (Arabic) and a non-inverted language (Hebrew), they have developed the ability to process both orders equally well. It is important to note that previous studies that investigated the effect of the structure of number–words presented the experimental verbal stimuli in written form on a computer screen. Our study is the first to present participants with aurally presented addition problems without including a written representation (verbal or numeric).

Furthermore, unlike previous studies, where participants were asked to type a numerical answer or verbally answer an addition problem, the participants in the current study were asked to decide whether the problem they heard (question and answer included) was correct or incorrect. This might be an additional reason for differences found between our findings (particularly regarding the order of presentation) and those of previous studies.

In addition, the current study explored the permeability of numerical processing to influences from the L1 and the L2 in highly proficient bilinguals. This issue is closely related to the debate questioning whether conceptual representations of bilinguals are exclusively shaped by the lexical structure of L1, or whether they are open to influences from a later learned L2 (e.g., Jiang, 2002; Degani et al., 2011). The current results suggest that the numerical processing of bilinguals might be shaped by exposure to two systems differing in the structure of number words, and not exclusively determined by the L1. Further, our bilingual participants were sensitive to the language of presentation, in that they showed different preferences in the L1 and in the L2, with the latter aligning closely with the performance of native speakers of the language.

The Arabic–Hebrew speakers in the current study differed significantly in the way they processed number words in Arabic from the way they processed number words in Hebrew. They were more sensitive to unit values when they heard problems recorded in Arabic but were more sensitive to decade values when they heard similar problems recorded in Hebrew. It is true that due to our methodological decision to present problem aurally, decade identity became available earlier in Hebrew whereas in Arabic, unit identity became available first. Of course, this could have been the cause of the observed pattern of results. However, the results could also be interpreted to mean that the structure of number words in the language influences the relative emphasis of unit and decades values in arithmetic performance. In accordance with this argument, in their study, Nuerk et al. (2005) concluded that decade distance seemed to determine responses in a number comparison task for English speakers, while overall distance was the most important predictor for German speakers, particularly when dealing with written number words.

Further Macizo et al. (2011), examined language influences by comparing Spanish/English and German/English bilinguals’ performance on a number comparison task. Their results demonstrate a reverse compatibility effect observed in the L1 Spanish task for the Spanish/English bilinguals (an expected pattern for a non-inverted language), and a regular compatibility effect observed in the L1 German task for the German/English bilinguals (an expected pattern for an inverted language). However, a reverse compatibility effect was observed in the L2 English task for both groups. Since their results suggest that bilinguals process two-digit number words selectively in their L1 and L2, they concluded that bilinguals are influenced by the language of presentation and process numbers according to the structure of number words for each language. The current flexible pattern found for the Arabic–Hebrew bilinguals aligns with these results, and extends them further to aural–verbal presentation.

In summary, the use of number processing as a case study for the interactions between language and cognition in bilinguals, allowed us to clearly demonstrate two important findings: (1) the L1 does not exclusively shape the conceptual knowledge and cognitive representations, and (2) extensive exposure to an L2 can result in flexibility of representation and adaptability to different linguistic structures.

## Conflict of Interest Statement

The authors declare that the research was conducted in the absence of any commercial or financial relationships that could be construed as a potential conflict of interest.
